# Bayesian spatial analysis of socio-demographic factors influencing pregnancy termination and its residual geographic variation among ever-married women of reproductive age in Bangladesh

**DOI:** 10.1186/s12889-020-09401-1

**Published:** 2020-09-04

**Authors:** Rifat Zahan, Cindy Xin Feng

**Affiliations:** 1 Department of Computer Science, University of Saskatchewan, 176 Thorvaldson Building, Saskatoon, S7N 5C9 Saskatchewan Canada; 2 Department of Community Health and Epidemiology, Faculty of Medicine, Dalhousie University, 5790 University Avenue, Halifax, B3H 4R2 Nova Scotia Canada; 3 School of Public Health, University of Saskatchewan, 104 Clinic Place, Saskatoon, S7N 2Z4 Saskatchewan Canada; 4 School of Epidemiology and Public Health Faculty of Medicine, University of Ottawa, 600 Peter Morand Cres, Ottawa, K1G 5Z3 Ontario Canada

**Keywords:** Pregnancy termination, Reproductive health, Spatial analysis, Bayesian logistic regression, Bangladesh

## Abstract

**Background:**

Unsafe pregnancy termination is a major public health concern among reproductive-aged women in many developing countries. This study evaluated the socio-demographic characteristics, as well as residual spatial correlation in pregnancy termination among Bangladeshi women.

**Methods:**

Secondary data was obtained from the Bangladesh Demographic and Health Survey for the survey year 2014. Data included 17,863 samples of ever-married women between the ages of 15-49 years, which is a national representative sample in Bangladesh. Bayesian spatial logistic regression was used to assess the associations between socio-demographic characteristics and pregnancy termination. We flexibly modeled the non-linear effects of the continuous covariates while accounting for residual spatial correlation at the district level.

**Results:**

Our findings revealed that about 19% of the respondents in Bangladesh reported ever had a pregnancy terminated. The risk of pregnancy termination was higher among women who had been working, had a higher wealth index, were in a conjugal relationship, had no children, were older and started their cohabitation earlier. Residual spatial patterns revealed the areas at a higher risk of pregnancy termination, including Panchagarh, Habiganj, and Sylhet after adjusting for covariates.

**Conclusions:**

Prevalence of pregnancy termination remains considerably high in Bangladesh. The study revealed significant associations of women’s age at survey time, age at first cohabitation, occupational status, socio-economic status, marital status and the total number of children ever born with reporting having a history of terminated pregnancy among Bangladeshi ever-married women. The identified socio-demographic characteristics and districts at an increased likelihood of pregnancy termination can inform localized intervention and prevention strategies to improve the reproductive healthcare of women in Bangladesh.

## Background

Many of the reproductive-aged women are at risk of unwanted pregnancies and therefore may seek ways to terminate pregnancies [1]. Pregnancy termination can happen in the form of abortion, miscarriage, menstrual regulation and stillbirth. Between the year of 2010 and 2014, about 55.7 millions termination of pregnancies in the form of abortions occurred annually worldwide [2], where 88% of them were from developing countries. In 2015, about 2.62 million pregnancies ended in stillbirths worldwide, where developed countries comprised about 1.8% of such stillbirths, and, the rest of them were from developing countries [3]. Bangladesh placed 7th by having 83 thousand stillbirths alone in 2015 [3]. In 2014, about 1,194,000 induced abortions occurred in Bangladesh [4], whereas, 430,000 menstrual regulations (MR) were performed nationwide at the same year [5]. Unsafe termination of pregnancy is one of the determinants of maternal health complexity [6], maternal mortality, and morbidity [7] and is a significant public health concern in many developing countries [8].

Bangladesh, predominantly being a patriarchal [9] and Muslim society [10], restricts women’s mobility and decision making. Specially, in the situation of family planning (i.e., spaces between pregnancies, number of total pregnancies, pregnancy termination, etc.), a husband’s or partner’s decision influences the pregnancy outcome [9]. In Bangladesh, pregnancy termination has puzzle pieces of legal, religious and moral grounds [10]. In the situation of unwanted pregnancies, women usually rely on informal or traditional methods for pregnancy termination, which includes homeopathic, allopathic medicines, oral contraceptives, herbs, plant roots, drinking hot salt water, etc. [9]. Abortion is not legal [10] for any purpose other than saving a woman’s life [5] or under the guise of menstrual regulation [11]. Women face barriers in accessing pregnancy termination services [9]. The Sustainable Development Goal (SDG) emphasizes the need to ensure universal access to sexual and reproductive health-care services, for family planning, information and education, and the integration of reproductive health into national strategies and programs, achieve gender equality and empower all women and girls to make the decision about their reproductive health [12, 13]. Many of the pregnancy terminations in Bangladesh are performed by untrained service providers in unsafe conditions [5], which may lead to maternal health complexity [6] and mortality [7]. Given these serious consequences for pregnancy termination, understanding its risk factors is essential to help policymakers to develop targeted prevention and intervention strategies for reducing pregnancy termination.

Previous studies have identified socio-demographic factors that are associated with pregnancy termination in developing countries. For example, age of the women at the survey time, marital status, education level, employment status and total number of children have were reported to be significantly associated with an increased risk of pregnancy termination [14–22]; however, the research findings in terms of the direction and the strength of the associations have been mixed. In the context of Bangladesh, only a few studies examined the risk factors associated with pregnancy terminations at some specific regions [23–25], which found older women with lower education are at an increased risk of having pregnancy termination. Nevertheless, no nationwide, population-based study research has been conducted to examine the extent to which different socio-demographic factors are associated with pregnancy termination.

Further, the social context in which the mothers are embedded may also play a role for influencing on their behaviors and health outcomes, according to the social disorganization theoretical framework [26, 27]. Neighbourhood or communities that have strong social ties and cohesion could provide opportunities to fulfil mothers’ physical, psychological, and social developmental needs, which may reduce the likelihood of unplanned or unwanted pregnancies. By contrast, socially disorganized neighbourhoods, which are characterized by social and economic disadvantage, may offer few reproductive and maternal health resources that normally help mothers to develop the physical, social, and emotional competencies necessary for reach their full potential for health and well-being. Moreover, if areal-level contextual factors contribute to the risk of pregnancy termination, then differential risk should be expected within a large geographical area in which variation in these factors is present. In this case, for women residing in the communities or neighbourhoods that are in close proximity to each other, we would expect some degree of spatial correlation in the risk of pregnancy termination at the neighbourhood level after adjustment for individual-level characteristics known to be associated with pregnancy termination. If an outcome is geographically dependent, ignoring such correlation could lead to incorrect inference. Therefore, a spatial analytical approach is useful for incorporating the geographical information of terminated pregnancies. Bayesian spatial models using Integrated Nested Laplace Approximation [28] provides a fast computation for fitting Bayesian spatial models, which allows incorporating spatial random effects for taking into account the effect of unobserved areal level covariates.

Some recent studies applied Bayesian spatial modeling to identify the risk factors while uncovering the residual spatial pattern in the disease risk. For example, the Bayesian spatial logistic regression model was applied to examine the association of socio-demographic and spatial variation in diabetes and hypertension in Bangladesh [29]. Another study used a structured Bayesian spatial logistic regression model to assess the socio-demographic and geographic inequalities in under- and over-nutrition among children and mothers [30]. Despite the spatial analyses conducted in literature, there has been limited attempt to use of spatial analysis techniques to analyze geographical patterns of pregnancy termination. This study attempts to contribute to this literature by modeling the residual spatial effect of pregnancy termination after accounting for the individual-level characteristics. Uncovering the high risk areas of pregnancy termination can be very informative for designing localized intervention and prevention strategies. Furthermore, the findings of this study could guide policymakers in designing effective public health interventions to reduce pregnancy termination-related maternal morbidity and mortality. The objectives of this paper are to examine the prevalence of pregnancy termination and identify socio-demographic characteristics as well as residual spatial correlation in pregnancy termination among women in Bangladesh based on nation-wide population-based data.

## Methods

### Study data

The present study used the Bangladesh Demographic and Health Survey (BDHS) from the survey year 2014. A total of 17,863 ever-married women between 15 and 49 years of age were interviewed [31]. Their marital status at the time of the survey can be married, separated, divorced or widowed. The survey was conducted under the authorization of Bangladesh’s National Institute of Population Research and Training (NIPORT) of the Ministry of Health and Family Welfare using a two-stage stratified sampling design. At the administrative level, Bangladesh was divided into 7 divisions and 64 districts (zilas) at the time of the survey. The 64 districts (zilas) are considered to examine the spatial variation in the termination of pregnancies. Data collected include participants’ socio-demographic profile, family structure, and pregnancy-related information [31]. The shape file of Bangladesh administrative level 2 (i.e., district-level) was downloaded from the link: https://gadm.org/download_country_v3.html, which is freely available for academic use and other non-commercial use. R [32] package rgdal[33] was used to read the shape file, and R package ggplot2 [34] was used to generate the maps following regression analysis.

### Outcome variable

The dependent variable employed for this study was “pregnancy termination” which was derived from the question “Have you ever had a pregnancy that miscarried, ended using menstrual regulation, was aborted, or ended in a stillbirth?” and responses were coded 0 = “No” and 1 = “Yes” [31].

### Explanatory variables

Explanatory variables considered in the analysis were identified from the literature [21–25, 35], which included: age of the women at survey time, age at first cohabitation, education level (no education, primary, secondary, and higher education), occupation (including professional, clerical, sales, agriculture, household services, skilled/unskilled manual jobs), religion (Muslim, Buddhism, Christianity, Hinduism, and others), place of residence (rural vs. urban), marital status (married, separated, divorced, and widowed) of the woman, number of children ever born, number of alive children. The current partner/husband’s level of education and occupation were also considered. We also considered the wealth index as an explanatory variable, which measured household inequalities. In Demographic and Health Survey (DHS), wealth indices are derived through principal component analysis based on household-level ownership of various assets (e.g. bicycles, cars, radios) and on housing characteristics (e.g. flooring material, drinking water source, type of toilet facility). The first principal component is taken as the underlying index of wealth. Each household is assigned a weight or factor score and the resulting asset scores are standardized in relation to a normal distribution with a mean of zero and a standard deviation of one [36]. The wealth quintiles (from lowest to highest) are then obtained by ranking each person in the population by his or her score and then dividing the ranking into five equal categories, each comprising 20% of the population.

### Statistical analyses

Multicollinearity among all the covariates was evaluated using generalized variance inflation factor (GVIF) [37], which is a generalization of the variance inflation factor (VIF). GVIF is applicable to measure the collinearity among covariates, such as dummy regressors from a polytomous categorical variable by considering the size of the joint confidence region for the related coefficients. Literature suggests reporting GVIF^1/(2·*df*)^, where *df* is the number of dummy variables in a categorical variable, is analogous to reporting the square root of the VIF for a single coefficient [37]. As a rule of thumb, VIF of 2.5 (i.e., $\sqrt {\text {VIF}}\approx 1.58$) or greater is a cause for concern for logistic regression.

Previous investigations on pregnancy termination are mostly based on logistic regression without accounting for geographical clustering [18, 38]. If the residuals of the regression model are independent and not spatial correlated, the model can be fitted using the frequentist survey logistic regression or generalized additive mixed effect models. However, in our study, mothers residing in nearby regions were often exposed to unmeasured similar social environment and health services access, so we suspect there might exist spatial correlations across different communities. Bayesian methodology, using Markov chain Monte Carlo (MCMC) methods, has become the predominant choice for spatial analysis to account for spatial correlation. However, it is widely acknowledged that MCMC techniques can be computationally very demanding. Recently, integrated nested Laplace approximation (INLA) [39] has emerged as a computationally efficient alternative to MCMC methods for performing Bayesian analysis.

To evaluate the potential spatial dependence of pregnancy termination, we applied INLA using the 64 districts as the areal unit of the analysis. The so-called convolution random-effects model [40] includes two random-effects terms, an unstructured, independent random effect (*h*_*j*_) assigned for each district *j*, and a spatially structured random effect (*b*_*j*_). We model the latter as an intrinsic conditional autoregression (CAR) for each district, which borrows information from “neighboring” districts to produce more reliable estimates across all the districts. We considered a neighborhood structure in which two districts are considered neighbors if they share boundaries. The spatial logistic regression model can be expressed as

1 $$\begin{array}{@{}rcl@{}} \text{logit}\left(p_{ij}\right) = \boldsymbol{Z}^{T}_{ij}\boldsymbol{\beta}+s\left(t_{ij}\right)+\phi\left(t'_{ij}\right)+b_{j} +h_{j}, \end{array} $$

where *p*_*ij*_ denotes the probability of pregnancy termination for the *i*th individual residing in district *j*, $\boldsymbol {Z}^{T}_{ij}$ is the design matrix for the fixed effects covariates, ***β*** represents a vector the regression coefficients, *t*_*ij*_ denotes the age of the women at survey time and $t^{\prime }_{ij}$ denotes the age at first cohabitation for the *i*th individual residing in district *j*.

In the following, we describe random walk (RW) priors for the age of the women at survey time *s*(*t*_*ij*_). The RW priors for the age at first cohabitation *ϕ*(*tij*′) can be defined analogously. For simplicity of notation, let *s*_*t*_ denote *s*(*t*_*ij*_). RW of first order (RW1) is modelled as an unknown smooth function of the *k*th ranked value of *t* in ascending order, *t*_*k*_, *k*=1,⋯,*K*. Then,

2 $$\begin{array}{@{}rcl@{}} &&p\left(s_{t_{1}}\right)\propto \text{const} \end{array} $$

3 $$\begin{array}{@{}rcl@{}} &&s_{t_{k}}|s_{t_{k-1}}\sim \text{Normal}\left(s_{t_{k-1}}, \tau_{s}\right), \, \, k=1, \cdots, K. \end{array} $$

For random walks of second order (RW2), we assume

4 $$ p\left(s_{t_{1}}\right)=p\left(s_{t_{2}}\right)\propto \text{const}\\  $$

5 $$ {{}\begin{aligned} &&s_{t_{k}}|s_{t_{k-1}}, s_{t_{k-2}}\!\sim\! \text{Normal}\left(2s_{t_{k-1}}\,-\,s_{t_{k-2}}, \tau_{s}\right), \, \, k\!\!=3, \cdots, K, \end{aligned}}  $$

where *τ*_*s*_ is a precision parameter, which is the inverse of the variance parameter $\sigma ^{2}_{s}$. The higher the precision, the smoother the estimated parameter vector. By default, a non-informative logGamma prior is assumed on the logarithm of the precision, which is equivalent to assume a Gamma prior on the precision, i.e., Gamma (*a*_1_,*a*_2_) with mean *a*_1_/*a*_2_ and variance $a_{1}/a_{2}^{2}$. The values used for the default prior for INLA are *a*_1_=1 and *a*_2_=10^−5^. The choice of *a*_1_=1 for the shape parameter of the gamma reduces the prior to a simple exponential distribution. The small value of *a*_2_ means that the prior for the precision as both a large mean and variance.

This spatial dependence is formalized by imposing the ICAR prior distribution on the structured spatial random effects,

6 $$\begin{array}{@{}rcl@{}} b_{j}|b_{j'\neq j}\sim \text{Normal}\, \left(\bar{b}_{j}, \sigma^{2}_{b}/m_{j}\right), \end{array} $$

where $\bar {b}_{j}$ denotes the mean of the spatial random effects of the neighborhoods, and *m*_*j*_ denotes the number of neighbors of district *j*. We model the spatially unstructured random effect *h*_*j*_ as an independent and identically distributed (IID) normal random variable $\text {Normal}\left (0, \sigma ^{2}_{h}\right)$. The formulation of the spatial model allows us to simultaneously estimate the residual spatial dependence and investigate the impact of various predictors related to pregnancy termination. The random effect terms can be interpreted as the effect of the district of residence on the pregnancy termination on each individual.

The priors for the precision $\tau _{b}=1/\sigma ^{2}_{b}$ and $\tau _{h}=1/\sigma ^{2}_{h}$ assigned with a non-informative Gamma prior Gamma (1,10^−5^). We assign vague priors for ***β***∼Normal (0,10^6^). We selected the non-informative priors for the parameters and their variance components, which allowed the observational data to have the greatest influence on posterior distributions without being greatly affected by the settings of priors.

Statistical software package R 3.5.0 [32] is used to conduct all the analysis. R-INLA package [39] provides reliable estimates within fast computation time to analyze spatial model in the Bayesian context [41]. Each survey respondent was given a sample weight provided by the BDHS to account for the unequal likelihood of selection and non-response. To ensure that our findings are representative of the Bangladesh population, the sample weights were incorporated in the analysis by specifying the option ‘weight’ as the sampling weight in the inla function, which scales log-likelihood by multiplying weights and individual log-likelihood [39]. Therefore, individual likelihood function increases with increased weight.

To select a parsimonious model, we firstly include all the covariates in the model and then select the best covariance structure among models without district level random effect, denoted as NO, model with only an IID random effect (*h*_*i*_), model with only a CAR (*b*_*i*_), and model with both CAR and IID random effects (*b*_*i*_+*h*_*i*_). After a covariance structure is determined, manual backward model selection was conducted to select the best set of fixed-effects covariates. All the variables were tested for significant interactions. Any individuals having missing data for the selected covariates were removed before regression analysis.

To compare various models, we employ the Watanabe-Akaike Information Criterion (WAIC) [42]. WAIC is invariant to parametrization and also works for singular models, which can be interpreted as a computationally convenient approximation to cross-validation and is defined as WAIC=LPD+*pD*, where LPD is the expected log pointwise predictive density and *pD* is the estimated effective number of parameters [42]. Models with smaller values of WAIC are preferred as they achieve a more optimal combination of fit and parsimony. As a rule of thumb, candidate models with a WAIC within 2 units of the ‘best’ model have a similar model fit, while models with a larger WAIC have a worse model fit [42].

## Results

### Characteristics of study participants

A total of 17,863 ever-married women between 15 and 49 years of age were interviewed [31]. Of those, 41 participants were excluded from our analysis due to the missing values in the response and covariates. The final sample of the analysis, therefore, included 17,822 respondents, of which almost one-fifth 19.44% (n=3,466) reported that they had terminated a pregnancy. The distributions of observations by pregnancy termination for each level of categorical predictors are displayed in Table 1.

**Table 1:** Socio-demographic characteristics of the study respondents

			Terminated Pregnancies			
	Total		Yes		No	
Covariate	Frequency	Column %	Frequency	Row %	Frequency	Row %
**Residence**						
(n = 17,822)						
Rural	11659	65.4%	2176	18.7%	9483	81.3%
Urban	6163	34.6%	1290	20.9%	4873	79.1%
**Respondent’s Education**						
(n = 17,822)						
No Education	4191	23.5%	878	21.0%	3313	79.1%
Primary	5215	29.3%	1014	19.4%	4201	80.6%
Secondary	6709	37.6%	1242	18.5%	5467	81.5%
Higher	1707	9.6%	332	19.5%	1375	80.6%
**Respondent’s Occupation**						
(n = 17,822)						
Not Working	12214	68.5%	2220	18.2%	9994	81.8%
Currently Working	5608	31.5%	1246	22.2%	4362	77.8%
**Husband/Partner’s Education**						
(n = 17,819)						
No Education	5050	28.3%	1014	20.1%	4036	79.9%
Primary	4843	27.2%	921	19.0%	3922	81.0%
Secondary	5255	29.5%	992	18.9%	4263	81.1%
Higher	2671	15.0%	537	20.1%	2134	79.9%
**Husband/Partner’s Occupation**						
(n = 17,745)						
Not Working	159	0.9%	33	1.0%	126	79.3%
Currently Working	17586	99.1%	3418	99.0%	14168	80.6%
**Wealth Index**						
(n = 17,822)						
Poorest	3241	18.2%	548	16.9%	2693	83.1%
Poorer	3353	18.8%	642	19.2%	2711	80.9%
Middle	3616	20.3%	680	18.8%	2936	81.2%
Richer	3755	21.1%	746	19.9%	3009	80.1%
Richest	3857	21.6%	850	22.0%	3007	78.0%
**Religion**						
(n = 17,821)						
Islam	16114	90.4%	3160	19.6%	12954	80.4%
Hinduism	1572	8.8%	287	18.3%	1285	81.8%
Buddhism	100	0.6%	14	14.0%	86	86.0%
Christianity	32	0.2%	5	15.6%	27	84.4%
Other	33	0.0%	0	0.0%	3	100%
**Marital Status**						
(n = 17,822)						
Married	16792	94.2%	3322	19.8%	13470	80.2%
Separated	239	1.3%	37	15.5%	202	84.5%
Divorced	169	1.0%	19	11.2%	150	88.8%
Widowed	622	3.5%	88	14.2%	534	85.9%
**Total Children Ever Born**						
(n = 17,822)						
No	1779	10.0%	196	11.0%	1583	89.0%
One	3963	22.2%	677	17.1%	3286	82.9%
Two	4707	26.4%	973	20.7%	3734	79.3%
Three	3322	18.6%	724	21.8%	2598	78.2%
Four or More	4051	22.7%	896	22.1%	3155	77.9%
**Total Children Alive**						
(n = 17,822)						
No	1885	10.6%	221	11.7%	1664	88.3%
One	4235	23.8%	730	17.2%	3505	82.8%
Two	5186	29.1%	1083	20.9%	4103	79.1%
Three	3347	18.8%	724	21.6%	2623	78.4%
Four or More	3169	17.8%	708	22.3%	2461	77.7%
**Age of the Respondent**						
(n = 17,822)						
	Median	IQR	Median	IQR	Median	IQR
Age at Survey Time	30.58	15.00	33.17	13.59	29.83	15.25
Age at First Cohabitation	15.92	3.66	15.75	3.87	15.92	3.58

As can be seen in Table 1, 65.42% of the respondents live in rural areas in Bangladesh. Approximately, 75% of the women had some form of formal education and 31.47% reported being involved in some form of occupation. More than 70% of the women reported that their husbands or partners have some level of formal education. About 99% of the women responded that their husbands were involved in some form of profession. Over 60% of the respondents were above the poverty line (i.e., having a middle or rich wealth index). The majority of the women were Muslims and married and had at least one child or had at least one child alive.

The median age of the respondents at the time of the survey was around 31 years and women started their conjugal life either with their husband or partner approximately at the median age of 16. The lowest age, when women started their conjugal life was when they were about 10 years old. About 75% of the women, who had terminated pregnancies started their conjugal life before reaching the age of 18, which is the legal age for women to get married in Bangladesh.

### Model selection

For examining multicollinearity among covariates, total children ever born and total children alive are of concern in this analysis with GVIF^1/(2·*df*)^ values equal to 2.50 and 2.49, respectively, which are greater than 1.58, as shown in Table 4 in the Appendix. We therefore excluded total number of children alive from the subsequently analysis.

All the rest of the covariates of interests were included in the model for selecting the best covariance structure. The model with CAR random effect gives lower WAIC as compared to IID and CAR+IID models, therefore, CAR was selected as the best covariance structure. Then, backward model selection was carried out for selecting the best set of fixed effect covariates. Residence, respondent’s occupation, wealth index, marital status, total number of children ever born, age at survey time and age at first cohabitation were selected as the covariates in the model.

To demonstrate the impact of misspecification of district level random effect structure and non-linearity of the effects for the age variables, we included the selected covariates after model selection and also considered modelling the continuous covariates (i.e., age of the women at survey time and age at first cohabitation) having linear effect vs. non-linear effect using RW1 or RW2. Table 2 presented the results of the model comparison according to WAIC, which indicated that there was within-district correlation in the risk of pregnancy termination, since Model NO performed much worse than the other models with district-level random effects yielding much higher WAIC score. Among all the competing models, modeling the age variables having linear effects consistently performed worse than the models imposing RW1 or RW2 structures for modeling the effects of age variables. Using RW1 for the age variables outperformed the counterpart models using RW2. Across all the models, CAR model with RW1 yielded the lowest WAIC, which was therefore selected as the final model. This suggested that including spatially structured random effects and random walk priors accounting for the non-linear effects of the continuous variables improved the predictive accuracies of the models.

**Table 2:** Comparison of model fits using WAIC

**Model**	WAIC (pD)
**Linear Effect**	
NO	17046.39 (26.03)
IID	16972.03 (90.09)
CAR	16968.59 (84.74)
CAR + IID	16971.54 (89.30)
**RW1**	
NO	16869.55 (153.11)
IID	16791.42 (217.95)
CAR	16788.28 (213.43)
CAR + IID	16790.76 (217.74)
**RW2**	
NO	16902.64 (41.39)
IID	16826.31 (104.54)
CAR	16823.50 (99.81)
CAR + IID	16826.07 (104.23)

### Sensitivity analysis

A careful prior sensitivity analysis was conducted to assess the robustness of the Bayesian inference. If the posterior distribution dramatically changes when the base prior parameter values are altered slightly, the Bayesian inference is not reliable. This analysis included the following: (i) changing the prior distribution of the precision parameters to Half Normal, Half Cauchy, Half t, Uniform, Penalized Complexity (PC) prior; (ii) changing the hyper-parameters (*a*_1_,*a*_2_) of the default gamma prior for the precision parameter *τ* from (*a*_1_=1,*a*_2_=10^−5^) to (*a*_1_=0.001,*a*_2_=0.001) and (*a*_1_=0.1,*a*_2_=0.1). As shown in Table 5 in the Appendix, the influence of prior specifications of the precision parameters for the random effect terms in the best fitting model is negligible on the basis of posterior summaries for all the parameter estimates as well as the model fits. The estimates for the fixed effects covariates are nearly identical and changing the hyper-parameters of the gamma distribution had no impact on the results, so the results were not reported for the sake of brevity and clarity.

### Factors associated with pregnancy termination

Table 3 presents the parameter estimates of the NO, IID, and CAR models with age of women at survey time and age at the first cohabitation modeled as RW1. The results of CAR+IID model are not reported since CAR+IID and IID models yielded almost identical model fits as shown in Table 2. As shown in Table 3, the covariate effects of the IID and CAR models were almost identical, which indicates that misspecification of the correlation structure of the random effects at the district level did not have much impact on the estimation of the covariate effects. Nevertheless, the significant effects of the wealth index were more pronounced under the IID or CAR models in comparison to the model without district-level random effects, i.e., model NO. More specifically, under the model NO, only the richest wealth index level was positively related to the pregnancy termination; whereas, under the IID and CAR models, middle, richer and richest wealth index levels were all significantly related to pregnancy termination. This implies there was a significant within district correlation in the risk of pregnancy termination that should be incorporated in building the model in order to properly evaluate the fixed effects covariates.

**Table 3:** Adjusted Odds Ratio (OR) and the corresponding 95% confidence intervals (CI) assessing the risk factors associated with reporting pregnancy termination based on the NO, IID and CAR models with the age of the women at the time of the survey and age at the first cohabitation modeled as RW1. The posterior standard errors of the variance components of the random effects and the 95% CIs are also reported. (n=17,822)

	NO		IID		CAR	
Covariate	OR	95% CI	OR	95% CI	OR	95% CI
**Residence**						
Rural						
Urban	1.08	[0.98, 1.20]	1.08	[0.98, 1.20]	1.09	[0.98, 1.2]
**Occupation**						
Not Working						
Currently Working	1.18	[1.09, 1.28]	1.19	[1.09, 1.29]	1.19	[1.09, 1.29]
**Wealth Index**						
Poorest						
Poorer	1.06	[0.93, 1.19]	1.07	[0.94, 1.22]	1.08	[0.95, 1.23]
Middle	1.12	[0.99, 1.27]	1.17	[1.03, 1.33]	1.18	[1.03, 1.34]
Richer	1.13	[0.99, 1.28]	1.18	[1.03, 1.35]	1.19	[1.04, 1.36]
Richest	1.15	[1.00, 1.32]	1.25	[1.07, 1.45]	1.26	[1.08, 1.47]
**Marital Status**						
Married						
Separated	0.50	[0.34, 0.73]	0.50	[0.34, 0.72]	0.50	[0.34, 0.73]
Divorced	0.39	[0.22, 0.67]	0.37	[0.21, 0.64]	0.37	[0.21, 0.64]
Widowed	0.41	[0.32, 0.52]	0.41	[0.32, 0.53]	0.41	[0.32, 0.53]
**Total Children Ever Born**						
No Child						
One Child	0.99	[0.82, 1.19]	0.99	[0.82, 1.19]	0.99	[0.82, 1.19]
Two Children	0.79	[0.65, 0.97]	0.79	[0.64, 0.96]	0.79	[0.65, 0.97]
Three Children	0.73	[0.59, 0.99]	0.71	[0.58, 0.88]	0.72	[0.58, 0.89]
Four or More Children	0.65	[0.53, 0.82]	0.63	[0.50, 0.79]	0.63	[0.51, 0.79]
**Standard deviation of random effect(s)**						
IID (*σ*_*h*_)			0.25	[0.19, 0.34]		
CAR (*σ*_*b*_)					0.44	[0.33, 0.61]
Age at Survey Time	0.41	[0.26, 0.73]	0.42	[0.26, 0.73]	0.42	[0.26, 0.73]
Age at First Cohabitation	0.06	[0.03, 0.17]	0.06	[0.03, 0.18]	0.06	[0.03, 0.18]

The covariate effects estimated based on the best fitting model, i.e., the CAR model with the age of women at survey time and age at the first cohabitation modeled as RW1 are interpreted as follows.

**Fig. 1:**
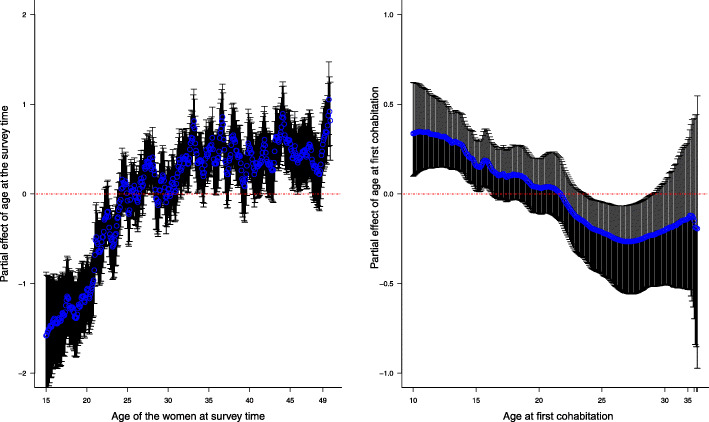
Partial effects of age at survey time (left panel) and age at first cohabitation (right panel) based on the best fitting model, i.e. CAR model adjusting all the covariates presented in Table 3. The blue points are the posterior mean estimates of the random effects for the age variables and the black vertical lines are the 95% point-wise confidence intervals. R [32] package ggplot2 [34] was used to generate the graphs
- The log odds of reporting pregnancy termination decreased with increased age at the first cohabitation and plateaued after about age 22 at the first cohabitation, as shown in the right panel of Fig. 1.

- The odds of reporting pregnancy termination did not differ significantly between women from urban versus rural regions (OR: 1.09; 95% CI [0.98, 1.20]).
- Women, who were involved in some form of occupation had 1.19 (95% CI [1.09, 1.29]) times higher odds of reporting pregnancy termination than for women, who were not working.
- The odds of reporting pregnancy termination increased with an increased wealth index of the women. Women in poorer, middle, richer and the richest socioeconomic status were approximately 1.08 (95% CI [0.95, 1.23]), 1.18 (95% CI [1.03, 1.34]), 1.19 (95% CI [1.04, 1.36]) and 1.26 (95% CI [1.08, 1.47]) times more likely to report ever having had a terminated pregnancy, respectively than women in the poorest socioeconomic status.
- Women, who were not in union (i.e., separated, divorced and widowed) were less likely to report having a history of terminated pregnancy compared to married women. More specifically, separated, divorced and widowed women were 0.50 (95% CI [0.34, 0.73]), 0.37 (95% CI [0.21, 0.64]) and 0.41 (95% CI [0.32, 0.53]) times less likely to report having a history of terminated pregnancy than married women.
- Women, who had at least one child were less likely to report having a history of terminated pregnancy compared to women who had no children. The odds of reporting pregnancy termination were 0.99 (95% CI [0.82, 1.19]), 0.79 (95% CI [0.65, 0.97]), 0.72 (95% CI [0.58, 0.89]) and 0.63 (95% CI [0.51, 0.79]) times less for women with one child, two children, three children, four or more children, than women who had no children.
- The log odds of reporting pregnancy termination increased sharply as the age of the women at survey time increased up until about 25 years old and then gradually plateaued, as shown in the left panel of Fig. 1.

### Spatial disparity in pregnancy termination

**Fig. 2:**
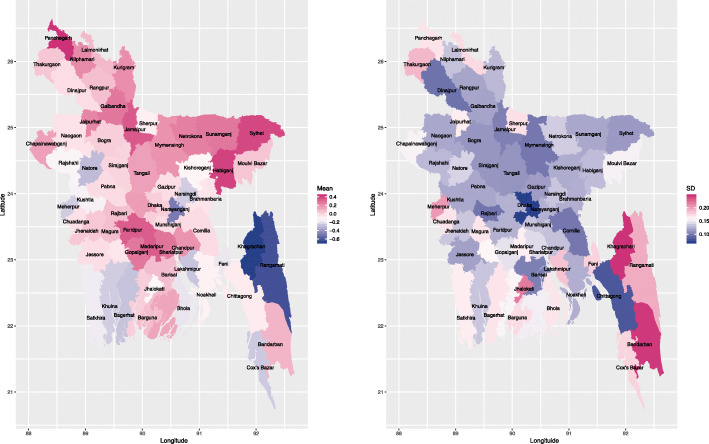
Posterior mean (left) and standard deviation (right) of the district-level random effect for the best fitting model, i.e. CAR model adjusting for all the covariates presented in Table 3. The shape file of Bangladesh administrative level 2 (i.e., district-level) was downloaded from the link: https://gadm.org/download_country_v3.html, which is freely available for academic use and other non-commercial use. R [32] package rgdal [33] was used to read the shape file, and R package ggplot2 [34] was used to generate the maps

The map of the posterior means for the district-level random effects based on the best fitting model, i.e. CAR model adjusting for all the covariates presented in Table 3 is given in the left panel of Fig. 2, which shows that the residual district effect was much higher in north and middle regions than the other regions. The right panel of Fig. 2 shows that the posterior standard deviations of the district-level random effects in the mainland area tend to be lower than the districts in the south-east. This demonstrates the within-district variation of pregnancy termination in the south-east tends to be higher than the rest of the districts after accounting for all the covariate effects. This is likely due to the different population sizes across the study region. More specifically, Rangamati, Khagrachari and Bandarban districts in the south-east are least populated districts in Bangladesh. In contrast, Dhaka and Chittagong districts are highly-densely populated. This may explain the observed variation in standard deviation.

**Fig. 3:**
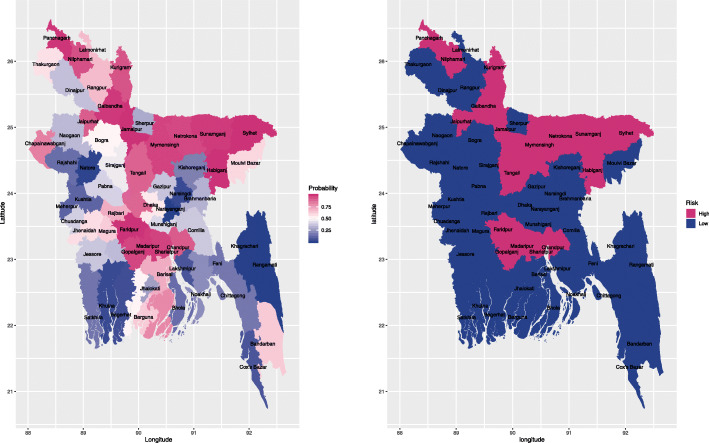
Posterior estimate of exceedance probabilities, defined as $q_{i}=p\left (e^{b_{i}}>1\right)$ (left) and districts with significantly elevated risk of reporting pregnancy termination [exceedance probability >0.90] (right), based on the best fitting model, i.e., CAR model adjusting for all the covariates presented in Table 3. The shape file of Bangladesh administrative level 2 (i.e., district-level) was downloaded from the link: https://gadm.org/download_country_v3.html, which is freely available for academic use and other non-commercial use. R [32] package rgdal [33] was used to read the shape file, and R package ggplot2 [34] was used to generate the maps

Identification of districts with significantly elevated residual risk for reporting pregnancy termination may help local public health authorities prioritize locations that require immediate interventions. We, therefore, used the posterior estimate of exceedance probabilities, defined as $q_{i}=p\left (e^{b_{i}}>1\right)$, which is an estimate of the probability of the spatial random effect exceeding the null value (*b*_*i*_=0). There were notable differences in the residual risk across the districts, as shown in the left panel Fig. 3. The exceedance probability in the north and middle districts had higher residual risk as compared to the other districts. The districts with significantly elevated residual risk of reporting pregnancy termination, i.e., exceedance probability greater than 0.90 were also presented in the right panel of Fig. 3, which revealed the districts with significantly elevated risk of reporting pregnancy termination after accounting for covariate effects.

To evaluate whether the spatial residual effect is dominating and the effect of the included covariates is only minor, we fit the CAR model without adjustment for the covariates. Our results showed that the CAR model without adjusting for the covariates yielded much worse model fit (WAIC=17361.18) as compared to the CAR model adjusting for the covariates (WAIC=16788.28). Comparing Fig 2 and Fig. 6, the spatial pattern of the random effect with and without adjusting for the covariates were similar, but not identical, which indicated that spatial variation can be explained by covariates to some extent.

**Fig. 4:**
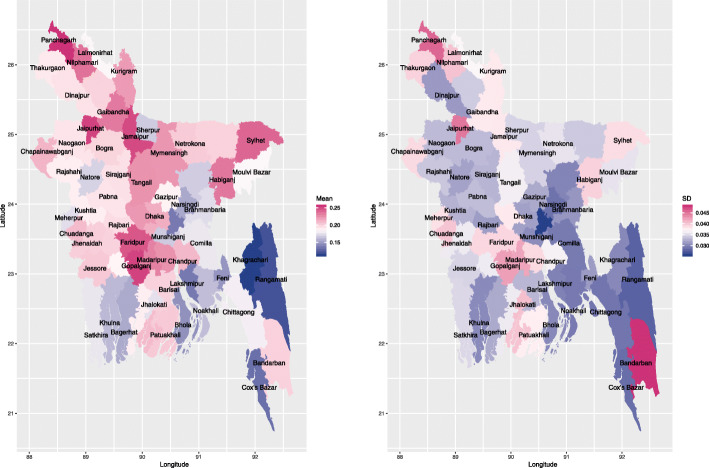
The left and right panels display the maps of the average and standard deviation of the posterior predicted probabilities of reporting pregnancy termination at each district, respectively, based on the best fitting model, i.e., CAR model adjusting for all the covariates presented in Table 3. The shape file of Bangladesh administrative level 2 (i.e., district-level) was downloaded from the link: https://gadm.org/download_country_v3.html, which is freely available for academic use and other non-commercial use. R [32] package rgdal [33] was used to read the shape file, and R package ggplot2 [34] was used to generate the maps

We further mapped the average of the posterior predicted probabilities of reporting pregnancy termination for all the women at each district, as shown in the left panel of Fig. 4. The identified areas for high risk of reporting pregnancy termination were at locations that were similar to the high-risk areas of residual district level effect as shown in the left panel of Fig. 2. The right panel of Fig. 4 depicted the standard deviation of the predicted probabilities of reporting pregnancy termination at each district, which showed that the variabilities of the probabilities of reporting pregnancy termination at the districts from the mainland areas were generally higher than those districts in the southeast except for the Bandarban district. This is different from the residual spatial pattern observed in the right panel of Fig. 2 due to accounting for the uncertainty in the covariate estimates, which confirms the importance of incorporating both covariates and spatial correlation using random effects in the model.

### Effect of misspecification of non-linear covariate effects

**Fig. 5:**
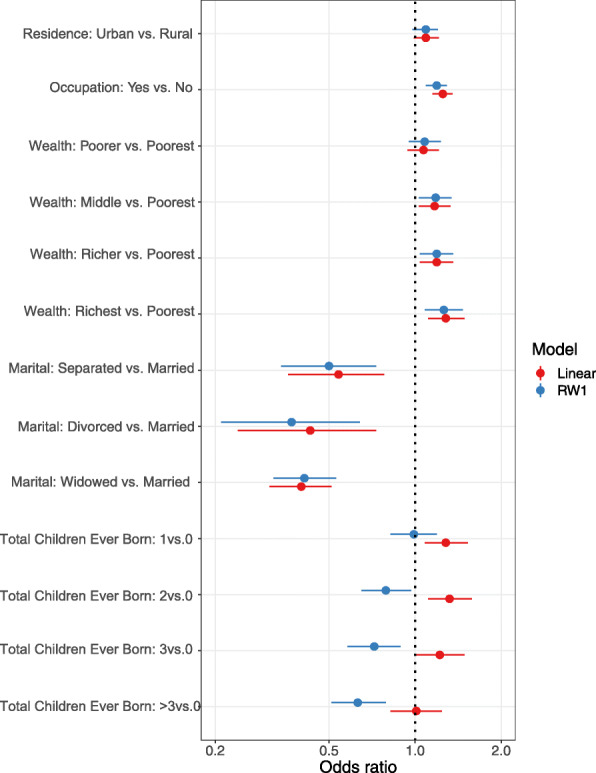
Comparison of adjusted odds ratios and 95% credible intervals of the fixed covariate effects based on the CAR models with linear versus non-linear (RW1) effects of age of women at survey time and age at first cohabitation

We also examined the impact of misspecifying the covariate effects by comparing the estimated regression coefficients of the CAR models for modeling the age variables having linear effect vs. non-linear effect using RW1. The forest plot of odds ratios (OR) and 95% confidence intervals were given in Fig. 5. As shown in the plot, the estimated covariate effects of the two models were mostly very similar, except for the total number of children ever born to the women. Under the CAR model with linear effects of the age variables, the odds of reporting pregnancy termination increased with an increased total number of children ever born to the women; whereas, a reversed association were observed for the CAR model with non-linear effects of age variables (i.e., RW1). This highlighted the importance of flexible modeling of the non-linearity of continuous covariates for properly drawing the inference of other covariates.

## Discussion

Based on the findings of this study, about one-fifth of ever married Bangladesh women had terminated the pregnancy. The findings of this study added to the limited literature on the reproductive health of women in Bangladesh. The study revealed significant associations of women’s age at survey time, age at first cohabitation, occupational status, socio-economic status, marital status, and the total number of children ever born with reporting having a history of terminated pregnancy among Bangladesh ever-married women.

The results from our study suggested that women who were involved in any occupation were at an increased risk of reported pregnancy termination in Bangladesh. This is consistent with another study conducted by in Ghanaian women based on Ghana DHS data [18]. There might be several reasons for employed women to experience terminated pregnancies compared to non-working women: decision-making power of working women regarding their reproductive health, prioritizing career goals over pregnancy, being more aware of contraceptive options including, menstrual regulation [18], or access to induced abortion through financial means [43] even though abortion is still illegal in Bangladesh. These may explain the reasons why working women tended to be more likely to have had voluntary termination of pregnancies. In contrast, working women may experience involuntary pregnancy termination for several reasons. Previous study suggested that working women had a higher risk of stillbirth [44]. Working women carry a higher risk of adverse reproductive outcomes (e.g., spontaneous abortion) [45] and some occupations are associated with high risk during pregnancy [46]. The authors suggested that parental exposure to harmful chemicals during their work may have an effect on the function and structure of the gamete, which may lead to an adverse outcome of pregnancy [45]. In a study conducted on Nepalese women, researchers found that women, whose major occupation was agriculture were more likely to report higher stillbirth [47]. Such literature explains both the voluntary and involuntary pregnancy termination among women. Since the outcome variable in our study considered stillbirths, miscarriage, menstrual regulations, and induced abortions, the findings from our study resemble the findings from previous studies. The findings from the current study stress that the workplace environment should be supportive of the need for the pregnancy of women.

The association of wealth index and pregnancy termination is complex, and research findings have been mixed. In some studies, no significant relationship was found between women’s socioeconomic status and ever having had a terminated pregnancy [15, 19]; whereas, other studies reported that the probability of having pregnancy termination was lower in the low socio-economic status of Brazillian and American women [14, 48]. On the other hand, another study reported that the probability of having terminated pregnancy was higher for women in high socio-economic status [35]. Our study showed that the risk of terminated pregnancies increased with the increased wealth index, which is consistent with the existing literature among Nigerian women based on Nigerian DHS data [49] and Ghanaian women based on Ghana DHS data [18]. Since induced abortion is still illegal in Bangladesh except under certain circumstances, accessing induced abortion or menstrual regulation may be unaffordable for women in low economic status [50]. Such a trend in pregnancy termination in terms of wealth index may be also explained by the increased financial power of women in higher socio-economic class [18].

The results available to date on studying the association between marital status and pregnancy termination have been also mixed. Some studies suggested that women not cohabiting with their husband or partner are at higher risk of termination of pregnancy compared to married women or women cohabiting with their partner [14–17]. However, in our study, we observed that being married increased the chance of having terminated pregnancies, which aligns with some of the other literature [18–20]. Research has shown that there is an association between intimate partner violence and pregnancy termination [51, 52]. Bangladesh has a high prevalence of intimate partner violence, which is associated with a high risk of pregnancy termination among Bangladeshi women [52]. Therefore, intimate partner violence might plausibly explain why married women are at a higher risk of pregnancy termination among our study respondents. In the recent BDHS survey, information on domestic violence was not well-documented. Therefore, drawing conclusions about the effect of domestic violence on pregnancy termination was not possible in the current study. Furthermore, a lower chance of pregnancy termination among separated, widowed or divorced women may be also attributable to their lower likelihood of getting pregnant as compared to married women [18].

We observed that the probability of reporting terminated pregnancies decreased with the increased total number of children ever born to women. This finding is consistent with the findings for Ghana and Mozambique [53], which suggests that women with no children were more likely to terminate a pregnancy as compared to women with parity 4 and above. One possible explanation is that the younger women may be more likely to have unmet need for family planning, so tendency abortion could be high among younger women [54]. Further, Bangladesh has secured a higher score in the happiness index compared to many other developed and developing countries in the world [55]. Being a parent is a significant part of life among Bangladeshi couples, and children are considered to be one of the main sources of happiness [55]. Children’s education and well-upbringing are considered to be an indicator of success in the families of Bangladesh [55]. Pregnancy and happiness were found to be strongly associated with each other [56]. Therefore, having the desire for more children may contribute to the low risk of pregnancy termination among women who already have a child.

This study suggested that women’s ages at first cohabitation play a role in the likelihood of reporting termination of pregnancies. Women starting their conjugal life after the age of 22 are at a lower risk of pregnancy termination. This finding aligns with previous studies which showed that early conjugal life has been identified as the risk factor for adverse health outcomes for both mothers and children [57, 58]. It is to be noted that for Bangladeshi women, the legal minimum age for marriage is 18. Child marriage and early conjugal life are associated with adverse outcomes of reproductive health, including high fertility, risk of unplanned pregnancies, and risk of child mortality [58]. Policy intervention should be in place to encourage women to start their conjugal life at a later age (preferably at 22 years).

This study examined the effect of women’s ages at the survey time on the likelihood of termination of pregnancies. The log odds of reporting terminated pregnancy increased as age at the survey time increased. This trend supports the findings from other literature [18, 38, 53, 54]. This finding is expected, as the age at survey time measures the risk time for a woman having a pregnancy termination, so the probability of having had a pregnancy termination is expected to be higher among “old” respondents than among “young” respondents. An alternative explanation of the observed trend is that older women may have achieved the desired family size, which may result in pregnancy termination [18, 59]. By contrast, some studies in Ethiopia, Ghana, Kenya and Nigeria reported that older women were significantly less likely to have an induced abortion when compared with younger women [60, 61]. One possible explanation is that younger women are likely to postpone childbearing for the pregnancy and early motherhood at that age due to the unmet need for contraception methods, which will lead to induced abortions [53]. Nevertheless, Bangladesh is a very conservative society, so pregnancy termination at younger age may be less likely to occur.

Our study did not find any significant relationship between woman’s education and termination of pregnancy, which is inconsistent with existing literature [53, 54, 62, 63]. More specifically, previous research found that educated women are less likely to have a history of pregnancy termination compared to uneducated women [62, 63]. One explanation is that women with lower education are less likely to use contraceptives to avoid unintended pregnancies that are likely to lead to abortion. On the contrary, other studies found that more educated women have a higher likelihood of induced abortion [53, 54]. One possible explanation is that women with higher education were likely to be in formal employment, who are therefore financially empowered and can afford to terminate a pregnancy as compared to poor women. Another possible explanation is that the employed women need to keep their formal employment status, which may lead to pregnancy termination. However, in our study, controlling for individual wealth level and age might have possibly explained the association between woman’s education and pregnancy termination in Bangladesh context.

To date, few studies have explicitly examined the spatial disparity of pregnancy termination by taking into account the geographical distribution of women. Our study revealed that the spatial models outperform the models without controlling for the spatial correlation, which suggested that impacts of geographical factors on pregnancy termination should be evaluated, and interventions should be formulated based on geographical features. More specifically, Panchagarh, Habiganj, and Sylhet were among the higher risk regions of terminated pregnancies after adjusting for covariates. Literature suggests that Panchagarh is one of the districts with a high rate of obstetric mortality in Bangladesh [64]. On the other hand, Khagrachari, Rangamati, and Narayanganj were among the lower risk regions and Kishoreganj, Rajshahi, Kushtia, and Jhalokathi districts had the lowest risk of pregnancy termination. The residual spatial patterns observed in pregnancy termination are likely due to unmeasured factors not represented in the models, which may be attributable to the differences in underlying socioeconomic, distance to health facilities and uneven allocation of public health resources.

### Policy implications

Our study has some policy implications: firstly, for working pregnant women, the workplace environment may be improved by modifying the condition of the workplace, providing support for work, by enforcing maternity leave regulation [65]. Secondly, policy could be in place to encourage young girls or women to start their conjugal life at a later age, preferably at 22 years in the form of financial incentives and/or by providing them empowerment program to delay their marriages [66]. Thirdly, healthy family planning programs could be introduced for women in conjugal relationships in Bangladesh. Lastly, the resulting prediction risk map may allow to measure the burden of the pregnancy termination at all locations, identify high-risk geographical areas for targeted interventions, and evaluate the impacts of intervention programs. This will be useful for the optimal utilization of scarce public health resources.

### Limitation of the study

The study has some limitations, which should be considered while interpreting the results. Firstly, past events have been reported based on recall of the respondents, such as pregnancy termination, age of first cohabitation and the total number of children ever born; therefore, the study may suffer from recall bias. Secondly, the BDHS data does not classify which of the terminated pregnancies were due to spontaneous or induced for therapeutic or elective reasons. Additional information on types of pregnancy termination should be collected to determine the extent to which the potential risk factors associated with different types of pregnancy termination. Thirdly, residential locations at the individual level are not available in DHS. The Bangladesh government makes a decision at the district level; therefore, the spatial analysis was conducted at the district level for drawing policy implications. Future studies could be conducted for fitting geostatistical models at finer spatial scales based on the GPS location of the center of each sampling cluster. Finally, this study has the inherent limitation of the cross-section study in the absence of information on the temporal relationship between the risk factors and the outcome. For example, the outcome variable i.e., ever experienced pregnancy termination, may occur before marriage. Nevertheless, Bangladesh is a very conservative society (i.e., patriarchal and predominantly Muslim) [9, 10]. The social norm does not support premarital sexual relationships or sexual relationships out of a marriage, and these are considered taboo. As a result, we suspect the likelihood of premarital pregnancies or pregnancy termination prior to marriage is very low. Additionally, a woman’s educational attainment, place of residence, employment status may be different during her pregnancy termination than at the time of the survey. The unobserved shift in these characteristics relative to the time-variant may bias the results of the analysis. Analytical studies, such as prospective cohort studies, seeking to establish relationships between risk factors and pregnancy termination, are therefore warranted to address the limitations of the cross-sectional study.

### Strength of the study

Despite the limitations, this study has some strengths. First of all, to the best of our knowledge, no studies have investigated unobserved spatial variation in pregnancy terminations while studying the impact of a range of demographic and socio-economic factors. Our study indicated that properly accounting for the geographical clustering and flexibly modeling the non-linear effects of the continuous covariates are crucial for drawing a valid statistical inference. In addition, this is a national-wide study, which provides the ground for policy-makers to employ policies to provide safer service for family-planning or reproductive health to women living in high-risk regions of terminated pregnancies.

## Conclusions

Our study revealed that women in Bangladesh, who had a higher wealth index, in a conjugal relationship, or had no children are at a higher risk of having terminated pregnancies. This study has underlined that the district-specific variation is substantial in modeling the risk of pregnancy termination. The results may help local public health authorities measure burden of the disease at all locations, identify geographical areas that require more attention to improve the reproductive health care by providing women with proper family planning method to avoid unintended pregnancies, if abortion was performed, or need-specific health-care and counselling, if the pregnancy ended up in miscarriage or stillbirth. Such changes will help to achieve two of the broader Sustainable Development Goals aiming for good health and well-being and gender equality.

## Appendix A: Supplementary materials

**Table 4:** Generalized variance inflation factor (GVIF) values for all the covariates

**Covariate**	GVIF	*df*	GVIF^1/(2·*df*)^
Respondent’s education	2.85	3	1.19
Respondent’s occupation	1.06	1	1.03
Respondent’s marital status	1.12	3	1.02
Husband/Partner’s occupation	1.01	1	1.00
Husband/Partner’s education	2.39	3	1.16
Religion	1.04	4	1.01
Wealth	1.87	4	1.08
Residence	1.29	1	1.14
Age at first cohabitation	1.36	1	1.17
Age at survey time	2.25	1	1.50
Total children ever born	1569.16	4	2.51
Total children alive	1460.50	4	2.49

**Table 5:** Posterior mean and 95% credible interval (CI) of the variance component of the random effect terms and WAIC values for the CAR model with different prior specifications adjusting for all the covariates presented in Table 3

	Mean	95% CI	WAIC
**Gamma**			16788.28
Age at First Cohabitation	0.06	[0.03, 0.18]	
Age at Survey Time	0.42	[0.26, 0.73]	
District	0.44	[0.33, 0.61]	
**Half Cauchy**			16788.40
Age at First Cohabitation	0.06	[0.03, 0.18]	
Age at Survey Time	0.42	[0.26, 0.73]	
District	0.45	[0.34, 0.62]	
**Penalized Complexity**			16788.31
Age at First Cohabitation	0.06	[0.03, 0.18]	
Age at Survey Time	0.42	[0.26, 0.73]	
District	0.45	[0.34, 0.62]	
**Half Normal**			16788.13
Age at First Cohabitation	0.06	[0.03, 0.18]	
Age at Survey Time	0.42	[0.26, 0.73]	
District	0.47	[0.36, 0.65]	
**Half t**			16788.15
Age at First Cohabitation	0.06	[0.03, 0.18]	
Age at Survey Time	0.42	[0.26, 0.73]	
District	0.46	[0.39, 0.64]	
**Uniform**			16788.13
Age at First Cohabitation	0.06	[0.03, 0.18]	
Age at Survey Time	0.42	[0.26, 0.73]	
District	0.47	[0.36, 0.65]	

**Fig. 6:**
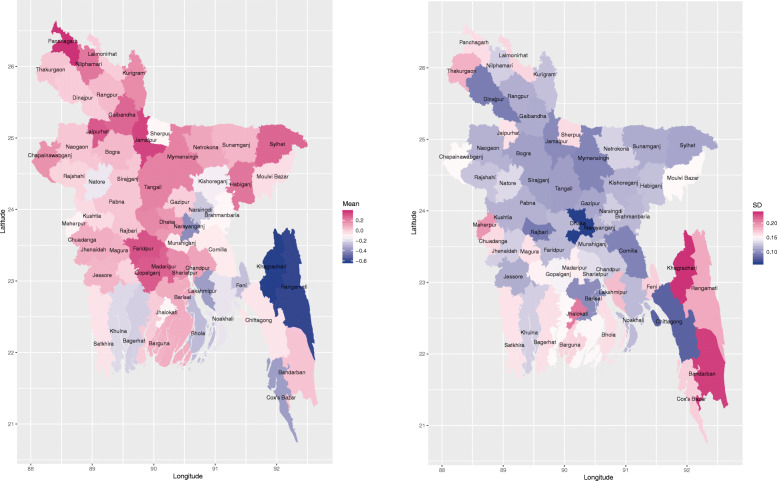
Posterior mean (left) and standard deviation (right) of the district-level random effect for the CAR model without any covariates. The shape file of Bangladesh administrative level 2 (i.e., district-level) was downloaded from the link: https://gadm.org/download_country_v3.html, which is freely available for academic use and other non-commercial use. R [32] package rgdal [33] was used to read the shape file, and R package ggplot2 [34] was used to generate the maps

## Data Availability

The data used in this study is available on this website following the approval of the DHS Program. https://dhsprogram.com/what-we-do/survey/survey-display-441.cfm.

## Abbreviations

BDHS: Bangladesh Demographic and Health Survey
CAR: Conditional Autoregressive
CI: Confidence Interval
DHS: Demographic and Health Survey
GVIF: Generalized Variance Inflation Factor
IID: independent and identically distributed
IQR: Interqartile Range
INLA: Integrated Nested Laplace Approximation
MCMC: Markov chain Monte Carlo
NIPORT: National Institute of Population Research and Training
OR: Odds Ratio
LPD: expected log pointwise predictive density
pD: Effective number of parameters
RW: Random Walk
VIF: Variance Inflation Factor
WAIC: Watanabe Akaike Information Criteria
